# Abnormal Intrapartum Cardiotocographic Tracing, Fetal Outcome and Placental Pathology

**DOI:** 10.3390/diagnostics16142224

**Published:** 2026-07-16

**Authors:** Eleonora Nardi, Simone Grassi, Andrea Costantino, Serena Simeone, Francesca Castiglione, Antonio Oliva, Vincenzo Arena

**Affiliations:** 1 Area of Pathology, Department of Laboratory and Hematological Sciences, Fondazione Policlinico Universitario A. Gemelli IRCCS, Università Cattolica del Sacro Cuore, 00168 Rome, Italy; eleonora.nardi@unicatt.it; 2 Forensic Medical Sciences, Department of Health Science, University of Florence, 50121 Florence, Italy; simone.grassi@unifi.it (S.G.); andrea.costantino1@unifi.it (A.C.); 3 High Risk Pregnancy Unit, Careggi University Hospital, 50121 Florence, Italy; serenasimeone09@gmail.com; 4 Section of Pathology, Department of Health Sciences, University of Florence, 50121 Florence, Italy; francesca.castiglione@gmail.com; 5 Section of Legal Medicine, Department of Health Surveillance and Bioethics, Fondazione Policlinico Universitario A. Gemelli IRCCS, Università Cattolica del Sacro Cuore, 00168 Rome, Italy; antonio.oliva@unicatt.it

**Keywords:** cardiotocographic tracing, placenta, histological examination, medico-legal issues

## Abstract

**Background:** Cardiotocography (CTG) represents a cornerstone in modern intrapartum fetal surveillance, allowing continuous assessment of fetal well-being through the analysis of fetal heart rate patterns in relation to uterine contractions. Despite its widespread clinical use, the interpretation of CTG tracings remains complex and is often associated with high interobserver variability and limited specificity in predicting adverse outcomes. In recent years, increasing attention has been directed toward placental pathology as a key determinant in the pathophysiology of adverse perinatal events. The placenta, as a dynamic organ mediating maternal–fetal exchange, plays a crucial role in fetal oxygenation and nutrient supply. Alterations in its structure and function may contribute to both chronic and acute fetal compromise. This retrospective study aimed to investigate the relationship between pathological intrapartum CTG tracings and maternal–fetal outcomes, with a particular focus on correlating these findings with macroscopic and microscopic placental abnormalities, compared to a control group of uncomplicated pregnancies with normal CTG patterns. **Material and methods:** We evaluated maternal, fetal, and placental histopathological data from 85 patients who exhibited pathological intrapartum cardiotocographic tracings. All deliveries occurred between January 2023 and March 2025 at the Obstetrics and Gynecology Departments of the ‘A. Gemelli’ University Hospital and Careggi University Hospital. A control group consisting of 50 women with normal CTG patterns and uncomplicated term singleton pregnancies, delivered at the same institutions, was used for comparison. **Results:** Cases with pathological CTG showed poorer neonatal outcomes compared with those with normal CTG. Infants in the pathological CTG group had lower Apgar scores, more frequent NICU admissions, and one case of hypoxic–ischemic encephalopathy. Mode of delivery, Apgar scores, NICU admission, and arterial pH were all significantly associated with CTG classification. A higher birth weight-to-placental weight ratio in the pathological CTG group suggested possible uteroplacental insufficiency. Macroscopic and histological placental findings, including hypercoiled umbilical cords and intervillous thrombosis, were also significantly more frequent in cases with pathological CTG. **Conclusions:** The findings of this study suggest that pathological CTG reflects not an isolated event but rather a multifactorial dysfunction of the feto-placental unit. Its associations with low Apgar scores, reduced arterial pH, abnormal BW/PW ratio, intervillous thrombi, and umbilical cord hypercoiling are consistent with existing evidence and support the interplay of chronic placental abnormalities and acute mechanical factors in the development of suspected fetal hypoxia. The future development of multivariate predictive models integrating clinical, biochemical, and anatomo-pathological variables may improve the early identification of pregnancies at risk and optimize intrapartum management, thereby reducing adverse perinatal outcomes.

## 1. Introduction

Cardiotocography (CTG) is a non-invasive monitoring technique widely employed in obstetrics, particularly during labor, to assess fetal well-being by simultaneously recording the fetal heart rate (FHR) and uterine contractions [1,2].

The underlying assumption is that variations in the FHR in relation to uterine activity reflect the fetus’s oxygenation status and may therefore serve as an early indicator of suspected fetal hypoxia [1].

Accordingly, CTG interpretation provides standardized frameworks that assist clinicians in the prompt identification of fetal hypoxia [3]. Despite its widespread use, CTG interpretation is challenged by considerable inter- and intra-observer variability, which can lead to inconsistent clinical decisions [4].

In this clinical context, placental pathology has gained increasing relevance in elucidating the mechanisms underlying major obstetric complications, including intrauterine growth restriction, preterm birth, stillbirth, and intrauterine infections [5,6].

The histopathological evaluation of the placenta is essential for assessing placental function throughout pregnancy, particularly in cases of obstetric complications [5]. It provides insight into the pathophysiological processes driving these conditions and contributes valuable information for interpreting maternal and neonatal outcomes in the postpartum period [5].

It is also important to emphasize that the interpretation of cardiotocographic tracings carries significant medico-legal implications, as such evaluations frequently constitute a central component of expert assessments in malpractice and legal dispute investigations. In particular, liability in obstetrics represents a critical issue in legal medicine, often associated with delayed caesarean section or failure to intervene in the presence of intrapartum complications [7]. In forensic cases, CTG tracings are commonly reviewed to determine whether earlier delivery might have been indicated. However, in the absence of objective and universally reliable clinical tools to confirm fetal hypoxia, such assessments remain largely retrospective and frequently rely on placental pathology findings [7].

The placenta is a transient and highly specialized organ of pregnancy that supports the normal growth and development of the fetus. Growth and function of the placenta are precisely regulated and coordinated to ensure the exchange of nutrients and waste products between the maternal and fetal circulatory systems [8]. Its strategic location at the fetal-maternal interface provides a record of pregnancy in which the cumulative effects of the intrauterine environment should be carefully examined.

Placental examination can be particularly helpful in investigating circumstances of significant medico-legal relevance, such as intrauterine death, stillbirth, spontaneous preterm delivery, and neonatal encephalopathy [5].

The main aim of this retrospective study was to correlate maternal and fetal outcomes, as well as macroscopic and microscopic placental findings, between the group of pregnancies in which the intrapartum CTG was pathological and a control group consisting of placentas from normal pregnancies without intrapartum cardiotocographic abnormalities.

## 2. Materials and Methods

### 2.1. Study Population

In this retrospective study, maternal and fetal clinical data, as well as placental histopathological findings, were systematically analyzed in a cohort of 85 patients who presented with pathological cardiotocographic tracings during labor, classified according to the International Federation of Gynecology and Obstetrics (FIGO) guidelines (2015) [2,9].

All patients delivered between January 2023 and March 2025 at the Department of Obstetrics and Gynecology of the ‘A. Gemelli’ University Hospital (Rome) and at the Department of Obstetrics and Gynecology of the Careggi University Hospital (Florence), both tertiary referral centers with high delivery volumes and standardized obstetric and neonatal care protocols.

The inclusion criteria were as follows:

- Singleton spontaneous pregnancy;
- Gestational age > 37 weeks;
- Absence of fetal malformations.

Pregnancies complicated by multiple gestations, preterm delivery, or fetal malformations were excluded to minimize potential confounding factors that could independently influence both cardiotocographic patterns and neonatal outcomes.

Regarding the indication for CTG monitoring, cardiotocography was initiated as a routine clinical practice.

The study group was compared with a control cohort of 50 patients with normal cardiotocographic intrapartum tracings and uncomplicated singleton term pregnancies, who delivered at the same institutions, matched for gestational age.

### 2.2. Data Collection

For both groups, detailed maternal demographic and clinical variables were collected, including maternal age, parity, obstetric history, and intrapartum management characteristics. Fetal and neonatal data included birth weight, Apgar scores, umbilical cord arterial pH, and the need for neonatal intensive care unit admission. In addition, all placentas underwent standardized macroscopic and microscopic examination by experienced pathologists, with particular attention to lesions associated with maternal and fetal vascular malperfusion, inflammatory processes, and umbilical cord abnormalities.

All these data were systematically entered into a dedicated database for statistical analysis.

### 2.3. Collection of Macroscopic and Microscopic Placental Examination Data

The placentas were collected, immediately after delivery, in the delivery room and fixed in 10% neutral buffered formalin. All specimens were subsequently processed and analyzed in accordance with the recommendations of the Amsterdam Placental Workshop Group Consensus Statement [6], ensuring standardized terminology and diagnostic criteria for placental lesions.

Sampling was performed by serial sectioning of the placental disc. This was followed by a detailed macroscopic examination, including assessment of placental weight, dimensions, shape, cord insertion site, membrane characteristics, and the presence of gross abnormalities such as infarctions, hematomas, or thromboses, in line with established pathological guidelines. Particular attention was paid to the umbilical cord, including its length, coiling index, and any structural anomalies.

Formalin-fixed, paraffin-embedded (FFPE) placental tissue samples were sectioned using a microtome at a thickness of 3–5 µm.

All histological slides were independently re-evaluated by three experienced pathologists (EN, FC, and VA), each blinded to the others’ assessments, in order to minimize observer bias and enhance diagnostic reliability.

In cases of diagnostic uncertainty or discordance, a collegial review process was performed to establish the final diagnosis.

Histological lesions were classified according to Benton et al. [10] (Section S1—Supplementary Material):

All observed lesions were recorded in the final histopathological report.

### 2.4. Statistical Analysis

All clinical and histopathological data were compared between patients with intrapartum pathological CTG tracings and those with normal intrapartum CTG. Categorical variables were analyzed using the Chi-squared test, while continuous variables were compared using Student’s t-test or analysis of variance (ANOVA), as appropriate.

A nominal logistic regression model was then performed to evaluate the simultaneous effects of maternal age, fetal/placental weight ratio, intervillous thrombosis, and umbilical cord hypercoiling on the CTG outcome (normal vs. pathological). The model’s goodness-of-fit was assessed using McFadden’s pseudo-R-square (R^2^(U)) and the Whole Model Likelihood Ratio Test. Effect significance was determined via Effect Likelihood Ratio Tests. Odds Ratios (OR) with 95% profile-likelihood confidence intervals (CI) were calculated to quantify risk. When a zero-cell count occurred in the contingency tables, standard finite Odds Ratios (OR) could not be mathematically computed. Statistical significance was set at α = 0.05. All analyses were conducted using JMP software (v. 19.0.4).

## 3. Results

Histopathological abnormalities were observed exclusively in cases with pathological cardiotocography.

Table 1 reports the main characteristics of both cases with pathological CTG and those with normal CTG. Statistically significant associations were identified across maternal and fetal variables, as well as macroscopic and microscopic findings from the placental examination (Table 2).

Among the maternal clinical data, a statistically significant association was found between the mode of delivery (cesarean section, vacuum extraction, spontaneous vaginal delivery) and CTG classification (normal vs. pathological), with a *p*-value < 0.0001.

Among the fetal outcome variables, statistically significant associations were observed for Apgar scores at 1 and 5 min (*p*-value: 0.01 and *p*-value: 0.03, respectively), admission to the neonatal intensive care unit (*p*-value: 0.01) and arterial pH (*p*-value: 0.03).

Among the variables correlating fetal well-being with macroscopic placental features, the birth weight-to-placental weight ratio was significantly higher in cases with pathological CTG compared to those with normal CTG (*p*-value: 0.03), suggesting a possible compromise in placental function. For the evaluation of this ratio, neonatal birth weight and the weight of the placental disc (excluding cord and membranes) were considered. The ratio between these two parameters was calculated and compared with reference percentile tables reported in the literature that relate placental weight and the birth weight-to-placental ratio according to gestational age [11].

Among the macroscopic placental findings, a significant association was observed between a hypercoiled umbilical cord (defined as >3 coils per 10 cm) and pathological CTG (*p* = 0.04).

Histological examination of the placental parenchyma revealed a significant association between intervillous thrombosis and pathological CTG, with a *p*-value of 0.03.

The overall multivariate model was statistically significant (Whole Model Test: χ^2^(4) = 14.29, *p* = 0.0064), demonstrating a strong goodness-of-fit with a McFadden’s pseudo-R-square (R^2^(U)) of 0.2339.

**Figure 1:**
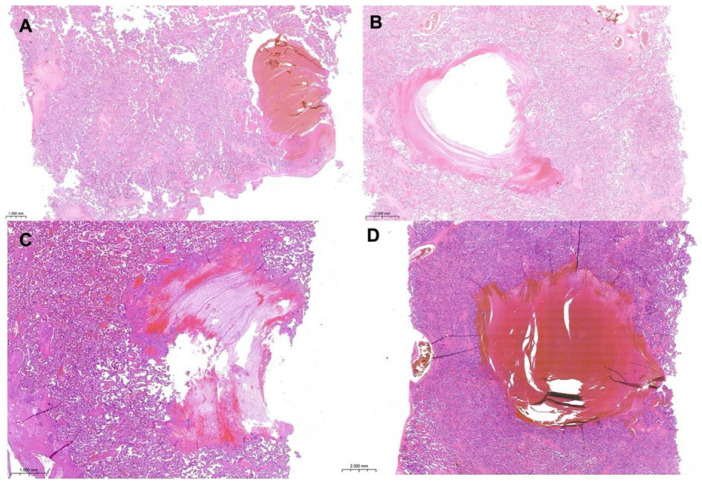
Histological sections of placental parenchyma with intervillous thrombosis (H&E (**A**): 2× magnification; (**B**): 4× magnification; (**C**,**D**): 10× magnification).

Among the investigated variables, intervillous thrombosis and hypercoiling (Iperspir) were identified as the only independent significant predictors of a pathological CTG:

- Intervillous thrombosis (Figure 1): Significantly associated with an increased risk of pathological CTG (Effect Test: *p* = 0.0323; 95% CI lower limit = 1.309).
- Hypercoiling (Iperspir): Significantly increased the likelihood of a pathological CTG outcome (Effect Test: *p* = 0.0379; 95% CI lower limit = 1.173).

Conversely, maternal age (*p* = 0.3772) and the fetal/placental weight ratio (*p* = 0.0798) did not achieve statistical significance within the multivariate framework, though a marginal trend was observed for the latter.

## 4. Discussion

The results of this study confirm that pathological CTG tracing during labor reflects a clinical impairment of fetal well-being, frequently related to alterations in utero-placental interplay and a consequent reduction in fetal oxygen reserve.

In forensic cases, CTG tracings are thoroughly analyzed to evaluate whether an earlier delivery, including operative intervention such as instrumental delivery or caesarean section, might have been indicated in order to prevent adverse neonatal outcomes.

However, in the absence of clinical tools capable of definitively demonstrating fetal hypoxia, forensic assessments remain inherently retrospective and are often limited by the subjective nature of CTG interpretation. As a result, these evaluations frequently rely on indirect evidence, among which placental pathology plays a central role. Histopathological examination of the placenta can provide objective insights into underlying conditions such as maternal or fetal vascular malperfusion, inflammatory processes, or thrombotic lesions, which may have contributed to impaired fetal oxygenation [7].

Placental pathology remains a controversial topic, with some authors strongly discouraging its use, while others consider the placenta a “black box” of the delivery process capable of providing valuable insights into perinatal outcomes [12,13].

In particular, placenta examination may help to clarify severe fetal outcomes, such as intrauterine deaths, stillbirths, and neonatal encephalopathy [5].

In the present study, we investigated whether a relationship exists between pathological CTG tracings and placental findings.

During labour, the primary clinical objective is the early detection of fetal hypoxia. However, due to the inaccessibility of the placenta during pregnancy, no direct tools are available to detect placental hypoxia [14]. For this reason, cardiotocography is widely used to monitor labor as fetal hypoxia is associated with characteristic variations in fetal heart rate [10]. Hypoxia during pregnancy may lead to fetal growth restriction and triggers adaptive responses in the placenta, including increased angiogenesis and reduced thickness of the vascular syncytial membrane [14]. Nevertheless, CTG has limited positive predictive value for fetal acidemia.

Therefore, placental pathology may provide valuable complementary information for confirming the clinical significance of non-reassuring tracings.

Currently, the relationship between CTG abnormalities during labor and placental microscopic findings remains poorly understood, and only limited evidence suggests a direct association [15].

In cases with pathological CTG, the birth weight-to-placental weight ratio was significantly higher (*p*-value: 0.03), with a slightly lower fetal weight suggesting a compensatory placental adaptation.

The placenta has the ability to modify its morphology and function in order to meet the nutritional demands required for optimal fetal growth [16,17].

Consequently, the birth weight–placental weight ratio is considered an indicator of placental efficiency [17].

Oxygen levels within the intervillous space increase between the first and second trimester, rising from approximately 20 mmHg to about 80 mmHg, and subsequently decrease during the third trimester [14].

That being said, the principal finding of our study is the statistically significant association between abnormal CTG patterns and specific placental microscopic abnormalities, particularly intervillous thrombi and vascular remodeling defects.

Of particular interest is the observed correlation between pathological CTG tracings and macroscopic morphological alterations of the umbilical cord (*p*-value: 0.04).

Hypercoiling of the umbilical cord, defined as more than three coils per 10 cm [10], represents a morphological condition associated with an impairment of fetal blood flow [18,19]. The degree of umbilical cord coiling, commonly quantified by the umbilical coiling index, is considered an important anatomical parameter reflecting the structural and functional integrity of the fetoplacental circulation. Excessive coiling may increase vascular resistance and predispose the cord vessels to compression, torsion, or kinking, particularly during uterine contractions, thereby compromising the efficiency of oxygen and nutrient exchange between the mother and the fetus [20].

This abnormality increases umbilical vascular resistance and the susceptibility of the cord to compression during uterine contractions, thereby promoting variable decelerations and non-reassuring fetal heart rate patterns [19]. From this perspective, hypercoiling may be considered a morphological marker of acute fetal risk.

An additional significant association was observed between pathological cardiotocographic patterns and the presence of placental intervillous thrombi.

These lesions, reflecting ischemic events and maternal malperfusion, impair maternal-fetal exchange and reduce the efficiency of fetal oxygenation [21,22].

Such alterations have also been reported to coexist with umbilical cord abnormalities, including hypercoiling, suggesting the involvement of concomitant haemodynamic and mechanical mechanisms in the pathogenesis of fetal hypoxia [23].

In the study population, the concomitant presence of umbilical cord hypercoiling, intervillous thrombi, and low fetal pH suggests a combined pattern of chronic maternal-fetal malperfusion and acute compromise of the fetoplacental circulation.

Vascular remodeling represents a fundamental process in placentation.

At the end of the first trimester, cytotrophoblasts remodel the uterine spiral arteries, transforming low-capacitance, high-resistance vessels into high-capacitance, low-resistance arteries that supply the superficial syncytiotrophoblast of the placental villi [14].

Interestingly, in our cohort of pregnancies with pathological intrapartum CTG tracings, gravidity and parity were relatively low.

This observation may be relevant because vascular remodeling is facilitated by the so-called “pioneering” changes of the spiral arteries and by the epigenetic memory of immune cells, which reflect residual modifications following a previous pregnancy [16,24].

More broadly, placenta vascular transformation is tightly regulated.

Defective remodeling, potentially associated with an imbalance between angiogenic and anti-angiogenic factors, may lead to preterm birth and ischemic placental diseases such as preeclampsia.

Conversely, excessive trophoblastic invasion may alter uterine artery vasomotor tone [14,25,26].

Together with reduced blood flow, these mechanisms increase the risk of thrombus formation [26].

Successful placentation depends on extensive remodeling of the uterine spiral arteries during early pregnancy, transforming high-resistance vessels into low-resistance, high-capacitance vessels capable of maintaining adequate uteroplacental perfusion throughout gestation and during the hypoxic stress of labor1. This process is regulated by complex interactions among trophoblasts, decidual immune cells, endothelial cells, and vascular smooth muscle cells [27,28].

An interesting interpretation of our findings is that previous pregnancies may induce a form of “placental memory” that enhances maternal adaptation in subsequent gestations. In multiparous women, prior trophoblast invasion may leave persistent structural, vascular, and immunological modifications within the uterus, facilitating more efficient spiral artery remodeling in later pregnancies [29,30]. In parallel, pregnancy-related immunological conditioning may promote a memory-like phenotype in decidual immune cells, improving trophoblast-endometrial interactions and vascular adaptation in subsequent pregnancies [31].

Consequently, women in their first pregnancy may lack this adaptive immunovascular conditioning. In primigravid patients, trophoblast invasion and placental vascular remodeling may therefore be less efficient, potentially reducing placental reserve capacity. Even subtle impairment of placental adaptation may compromise the fetus’s ability to tolerate the repetitive hypoxic stress associated with uterine contractions, thereby increasing susceptibility to pathological intrapartum CTG patterns.

This hypothesis is biologically plausible because abnormal fetal heart rate patterns during labor may represent the final manifestation of limited placental reserve rather than exclusively acute intrapartum events. A placenta that remains clinically compensated during pregnancy may become insufficient during labor, when uteroplacental perfusion physiologically decreases with each contraction. Therefore, the association between low gravidity/parity and pathological CTG tracings may reflect reduced placental adaptive reserve in first pregnancies and deserves further investigation.

In the analyzed sample, pregnancies characterized by pathological CTG tracings were also significantly associated with lower Apgar scores at both the first and fifth minutes of life (*p*-value: 0.03).

This relationship is well documented in the literature, where abnormal CTG tracings are interpreted as indicators of fetal heart rate alterations related to hypoxia or acidosis—conditions that compromise immediate neonatal adaptation [2,32].

Furthermore, studies by Low et al. [33] and Malin et al. [34] have demonstrated that an Apgar score lower than 7 at five minutes is strongly correlated with metabolic acidosis and an increased risk of neonatal neurological sequelae.

The findings of our study are consistent with this pathophysiological sequence and further support the role of CTG as an early and sensitive indicator of suspected perinatal compromise.

In this context, recent studies have shown that placental dysfunction, assessed indirectly by the Fetal Medicine Foundation preeclampsia screening algorithms, is associated with an increased risk of intrapartum fetal compromise in both induced and spontaneous labor, even in pregnancies without clinically overt preeclampsia. These findings support the concept that reduced placental reserve may impair fetal tolerance to the physiological hypoxic stress of labor, identifying placental dysfunction as a key pathophysiological substrate underlying intrapartum fetal compromise [35,36].

Overall, the variables significantly associated with pathological CTG patterns suggest the presence of two principal pathophysiological pathways underlying suspected fetal hypoxia.

The first involves a chronic placenta-mediated mechanism characterized by reduced placental efficiency, as indicated by an altered birth weight–placental weight ratio.

The second involves a mechanically driven pathway related to umbilical cord abnormalities, particularly hypercoiling, which may cause intermittent compression and acute ischemia. In addition, vascular lesions such as intervillous thrombosis may progressively reduce fetal oxygen reserve.

In our study, the histopathological features of the thrombotic lesions in the intervillous space were attributable to both a chronic and an acute event. However, the chronic nature of the thrombotic insult may also have contributed to the pathological nature of the cardiotocographic tracing.

Finally, the integration of detailed morphological analysis of the umbilical cord with advanced prenatal imaging techniques may represent a promising strategy for the earlier identification of structural and functional abnormalities potentially associated with suspected fetal hypoxia. In particular, parameters such as cord length, insertion site, coiling index, and the presence of true knots or vascular anomalies could be systematically correlated with prenatal ultrasound findings, including Doppler velocimetry of the umbilical arteries and vein. Moreover, advances in imaging modalities, including three-dimensional ultrasound and fetal magnetic resonance imaging, may provide additional insights into cord morphology and its spatial relationship with the fetus and placenta [37,38,39].

Such conditions may represent risk factors for increased fetal vulnerability to intrapartum hypoxic events, ultimately resulting in abnormal cardiotocographic patterns, lower arterial pH, reduced Apgar scores, and a higher likelihood of neonatal intensive care unit admission.

This study underscores the presence of an association between pathological CTG tracing and macroscopic and microscopic placental findings.

Summarized key points include:

- Pathological CTG tracings as indicators of impaired fetal well-being. Pathological intrapartum CTG tracings are associated with clinical signs of fetal compromise, supporting the concept that abnormal fetal heart rate patterns often reflect impaired fetal oxygenation and reduced placental reserve rather than isolated acute obstetric events. This finding is particularly relevant in both clinical and forensic settings, where CTG interpretation remains central to the assessment of intrapartum fetal status despite its known limitations in specificity and positive predictive value for acidemia.
- An important aspect emerging from the study is the role of placental histopathology as an objective complementary tool in the interpretation of non-reassuring CTG tracings. Since direct assessment of placental oxygenation during labor is not feasible, placental examination may provide retrospective evidence of chronic or acute conditions impairing maternal-fetal exchange, including vascular malperfusion, thrombosis, inflammatory and cord abnormalities.
- The significantly higher birth weight–placental weight ratio observed in pregnancies with pathological CTG tracings suggests altered placental efficiency and possible compensatory placental adaptation. Because the placenta can modify its morphology and vascular architecture in response to fetal metabolic demands, this ratio is widely considered an indirect marker of placental functional reserve.
- One of the most significant findings of the study is the association between pathological CTG tracings and macroscopic umbilical cord abnormalities, particularly hypercoiling. In fact, hypercoiling may impair fetoplacental circulation by increasing vascular resistance and enhancing susceptibility to compression or torsion.

This mechanical vulnerability may transiently reduce fetal blood flow, producing repetitive hypoxic episodes and characteristic variable decelerations on CTG monitoring.

- The study also demonstrated a significant association between pathological CTG patterns and placental intervillous thrombi. These lesions may impair oxygen diffusion and reduce placental exchange efficiency, progressively diminishing fetal oxygen reserve. Importantly, intervillous thrombi may represent both chronic and acute processes. Some lesions likely develop gradually during pregnancy as a consequence of impaired uteroplacental perfusion, whereas others may arise closer to labor in response to hemodynamic instability or vascular injury.

In particular, heir coexistence with umbilical cord abnormalities in the present study suggests that chronic placental insufficiency and acute mechanical compromise may act synergistically in the development of fetal hypoxia.

## 5. Future Perspectives

The results obtained by our study offer significant insights for clinical practice and future research. In particular, prospective studies with larger sample sizes and standardized, reproducible protocols will be essential to further explore and validate the combined predictive role of cardiotocographic parameters, biochemical indicators, and placental histological findings.

Future research should also focus on longitudinal study designs to better distinguish between chronic placental insufficiency and acute intrapartum events, clarifying their respective contributions to fetal compromise and neonatal morbidity. In this context, correlating dynamic intrapartum CTG changes with detailed postnatal placental examination may provide a more complete understanding of the temporal sequence of hypoxic injury.

## 6. Conclusions

The results of this study demonstrate that pathological intrapartum CTG may reflect a complex alteration of the feto-placental unit and it can be associated with adverse neonatal adaptation.

The observed associations with low Apgar scores, reduced arterial pH, altered birth weight–placental weight (BW/PW) ratio, intervillous thrombi, and umbilical cord hypercoiling support the hypothesis that both chronic placental factors and acute mechanical factors contribute to the pathogenesis of intrapartum suspected fetal hypoxia.

Looking ahead, the development of multivariate predictive models incorporating clinical, biochemical, and anatomo-pathological variables may improve the early identification of pregnancies at risk of suspected fetal compromise, thereby optimizing labor management and reducing possible adverse perinatal outcomes.

In forensic settings, the assessment of whether inadequate monitoring or delayed intervention—potentially leading to preventable fetal hypoxia—has occurred should be based on multiple sources of information, including placental pathology. However, not all placental abnormalities carry the same significance. In particular, abnormalities related to vascular remodeling and the presence of intervillous thrombi may represent the most relevant indicators of potential hypoxic stress, although they are not necessarily indicative of clinically or forensically significant events.

Placental pathology should not be regarded as a definitive “black box” of the delivery process. Rather, CTG findings, histopathological data, and clinical information must be interpreted in an integrated manner before establishing any causal relationship between a potential obstetric delay and an adverse perinatal outcome. In particular, routine histological placental examination after pathological CTG and measurement of the umbilical cord coiling index during macroscopic evaluation of the placenta could be suggested and recommended to try to better understand this association.

## Figures and Tables

**Table 1:** Main characteristics of cases with pathological and normal CTG.

Variable	Pathological CTG	Normal CTG
Mean gravidity	1	2
Mean parity	0	1
Mean 1 min Apgar	8	9
Mean 5 min Apgar	9	10
Mean fetal weight (g)	3258	3260
Mean placental weight (g)	473	558
NICU admissions	16	0
Long-term complications	1 (HIE)	0

**Table 2:** Significant maternal, fetal, and placental findings associated with pathological CTG.

	Parameter	*p*-Value (Pathological vs. Normal CTG)
Maternal clinical data	Mode of delivery (vaginal vs. c-section)	<0.0001
Fetal clinical data	Apgar score at 1 min	0.01
	Apgar score at 5 min	0.03
	Arterial pH	0.01
	NICU admission	0.03
Macroscopic placenta examination	Fetal/placental weight ratio	0.03
	Hypercoiled cord (>3 coils/10 cm)	0.04
Microscopic placenta examination	Intervillous thrombosis	0.03

## Data Availability

Data is contained within the article or Supplementary Material.

## Supplementary Materials

The following supporting information can be downloaded at: https://www.mdpi.com/article/10.3390/diagnostics16142224/s1, Figure S1: Macroscopic appearence of an hypercoiled umbilical cord; Table S1: Benton et al. Classification of histological lesions.

## Abbreviations

CTG	cardiotocographic tracing
FHR	fetal heart rate
BWPW ratio	birth weight/placental weight ratio
H&E	hematoxylin and eosin
FFPE	Formalin-fixed, paraffin-embedded
NICU	Neonatal Intensive Care Unit
