# Supplementary material for: Abnormal Intrapartum Cardiotocographic Tracing, Fetal Outcome and Placental Pathology
Source: Diagnostics (Basel). 2026 Jul 16;16(14):2224. doi: 10.3390/diagnostics16142224 (PMC13408046; doi:10.3390/diagnostics16142224)
Supplement: Supplementary file 1 [file diagnostics-16-02224-s001.zip › diagnostics-4304679-supplementary.pdf]

Section S1

Table S1: Benton et al. Classification of histological lesions

- Category 1:
  - evidence of maternal vascular malperfusion:
  - placental infarcts;
  - distal villous hypoplasia;
  - accelerated villous maturation;
  - increased syncytial knots;
  - villous agglutination.

- Category 2:
  - evidence of maternal decidual arteriopathy:
  - insufficient vessel remodelling;
  - fibroid necrosis.

- Category 3:
  - implantation site abnormalities:
  - microscopic accreta placentation.

- Category 4:
  - evidence of ascending intrauterine infections:
  - maternal inflammatory response;
  - fetal inflammatory response.

- Category 5:
  - evidence of placental villous maldevelopment:
  - chorangiosis;
  - chorangioma;
  - delayed villous maturation.

- Category 6:
  - evidence of fetal vascular malperfusion:
  - avascular fibrotic villi;
  - thrombosis;
  - intramural fibrin deposition;
  - villous stromal-vascular karyorrhexis;
  - stem villous vascular obliteration;
  - high-grade fetal vascular malperfusion.

- Category 7:

    - evidence of utero-placental separation:
    - chorionic hemosiderosis;
    - retroplacental hematoma.

- Category 8:
    - fibrinoid deposition:
    - increased focal/massive perivillous fibroid deposition.

- Category 9:
    - intervillous thrombi.

- Category 10:
    - evidence of chronic inflammation:
    - villitis of unknown etiology;
    - chronic intervillositis;
    - chronic plasmacell deciduitis;
    - chronic chorioamnionitis.

Section S2

Figure S1

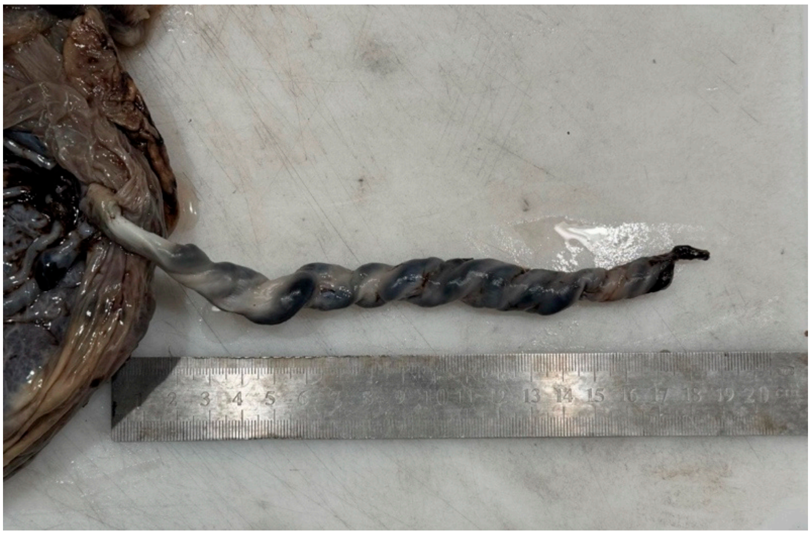

Supplementary material: macroscopic appearence of an hypercoiled umbilical cord.
